# Comparative transcriptome profiling of virulent and non-virulent *Trypanosoma cruzi* underlines the role of surface proteins during infection

**DOI:** 10.1371/journal.ppat.1006767

**Published:** 2017-12-14

**Authors:** A. Trey Belew, Caroline Junqueira, Gabriela F. Rodrigues-Luiz, Bruna M. Valente, Antonio Edson R. Oliveira, Rafael B. Polidoro, Luciana W. Zuccherato, Daniella C. Bartholomeu, Sergio Schenkman, Ricardo T. Gazzinelli, Barbara A. Burleigh, Najib M. El-Sayed, Santuza M. R. Teixeira

**Affiliations:** 1 Department of Cell Biology and Molecular Genetics and Center for Bioinformatics and Computational Biology, University of Maryland, College Park, Maryland, United States of America; 2 Centro de Pesquisas Rene Rachou, Fundação Oswaldo Cruz, Belo Horizonte, Minas Gerais, Brazil; 3 Departamento de Parasitologia, Universidade Federal de Minas Gerais, Belo Horizonte, Minas Gerais, Brazil; 4 Departamento de Bioquímica e Imunologia, Universidade Federal de Minas Gerais, Belo Horizonte, Minas Gerais, Brazil; 5 Departmento de Microbiologia, Imunologia e Parasitologia, Universidade Federal de São Paulo, São Paulo, São Paulo, Brazil; 6 Department of Immunology and Infectious Diseases, Harvard T. H. Chan School of Public Health, Boston, Massachusetts, United States of America; London School of Hygiene & Tropical Medicine, UNITED STATES

## Abstract

*Trypanosoma cruzi*, the protozoan that causes Chagas disease, has a complex life cycle involving several morphologically and biochemically distinct stages that establish intricate interactions with various insect and mammalian hosts. It has also a heterogeneous population structure comprising strains with distinct properties such as virulence, sensitivity to drugs, antigenic profile and tissue tropism. We present a comparative transcriptome analysis of two cloned *T*. *cruzi* strains that display contrasting virulence phenotypes in animal models of infection: CL Brener is a virulent clone and CL-14 is a clone that is neither infective nor pathogenic in *in vivo* models of infection. Gene expression analysis of trypomastigotes and intracellular amastigotes harvested at 60 and 96 hours post-infection (hpi) of human fibroblasts revealed large differences that reflect the parasite’s adaptation to distinct environments during the infection of mammalian cells, including changes in energy sources, oxidative stress responses, cell cycle control and cell surface components. While extensive transcriptome remodeling was observed when trypomastigotes of both strains were compared to 60 hpi amastigotes, differences in gene expression were much less pronounced when 96 hpi amastigotes and trypomastigotes of CL Brener were compared. In contrast, the differentiation of the avirulent CL-14 from 96 hpi amastigotes to extracellular trypomastigotes was associated with considerable changes in gene expression, particularly in gene families encoding surface proteins such as trans-sialidases, mucins and the mucin associated surface proteins (MASPs). Thus, our comparative transcriptome analysis indicates that the avirulent phenotype of CL-14 may be due, at least in part, to a reduced or delayed expression of genes encoding surface proteins that are associated with the transition of amastigotes to trypomastigotes, an essential step in the establishment of the infection in the mammalian host. Confirming the role of members of the trans-sialidase family of surface proteins for parasite differentiation, transfected CL-14 constitutively expressing a trans-sialidase gene displayed faster kinetics of trypomastigote release in the supernatant of infected cells compared to wild type CL-14.

## Author summary

Chagas disease, caused by the protozoan parasite *Trypanosoma cruzi*, is an infection that occurs in several Latin American countries, resulting in a mild illness or in severe damage of the heart and intestinal tract. Such a broad spectrum of clinical manifestations observed in Chagas disease patients is likely due to differences in host susceptibility as well as to a large heterogeneity among *T*. *cruzi* isolates. The identification of virulence factors that are differentially expressed in the parasite population is a valuable strategy for understanding of the distinct interactions that occur between this pathogen and its host, which may or may not lead to pathogenesis. By comparing the gene expression profiles of two *T*. *cruzi* strains that display contrasting virulence phenotypes in animal models of infection, we identified a central role for genes encoding surface proteins that is associated with the differentiation from intracellular replicative amastigotes to infective trypomastigotes. We showed that the expression of these genes occurs differentially within the two strains and this difference may be a factor that impacts parasite survival and dissemination in the mammalian host.

## Introduction

*Trypanosoma cruzi* is the etiological agent of Chagas disease, a disease affecting at least 8 million people throughout Latin America and for which there are only two drugs available, both with poor efficacy and harmful side effects (http://www.who.int/mediacentre/factsheets/fs340/en/). *T*. *cruzi* infection begins with metacyclic trypomastigotes released in the feces of a triatominae vector during a blood meal. After reaching the bloodstream through skin cuts or through the mucosa, trypomastigotes invade different cell types in the mammalian host. Once in the cytoplasm, they differentiate into replicative non-flagellated amastigotes, which undergo several rounds of division before differentiating again into trypomastigotes and bursting out of the host cell. Bloodstream trypomastigotes, ingested by the vector during a blood meal, differentiate into epimastigotes and replicate in the insect midgut [1]. Such a complex life cycle, involving several morphologically and biochemically distinct parasite forms and very different host environments, requires complex regulatory mechanisms to direct gene expression and the adaptive strategies that allow parasites to thrive within distinct hosts.

The sequence of the *T*. *cruzi* CL Brener genome, with an estimated haploid size of 55 Mb, revealed a highly repetitive genome with protein coding genes organized in long, unidirectional polycistronic transcription units [2]. Because of its hybrid nature and repetitive content, which prevented its complete assembly, the CL Brener genome is represented by two datasets of contigs, each corresponding to one haplotype [2,3]. The two haplotypes are referred to as “esmeraldo-like” or “non-esmeraldo-like” based on their parental strains [2,4]. Several genes, particularly members of large multicopy gene families, were excluded from the current assembly of the CL Brener genome, which is predicted to contain 41 pairs of chromosomes [3].

The naturally occurring *T*. *cruzi* population is comprised of a highly heterogeneous pool of strains with striking intra-species variation and exhibiting wide-ranging biological characteristics in experimental settings such as distinct morphology, growth rate, curves of parasitemia, virulence, sensitivity to drugs, antigenic profile, metacyclogenesis and tissue tropism. This variability may explain the wide-ranging pathogenesis observed in *T*. *cruzi* infections (reviewed by Buscaglia and DiNoia [5]). Those studies recently converged on a classification that proposes the existence of six major groups in *T*. *cruzi*, also known as discrete typing units (DTUs): I to VI [6]. Although clonal evolution is predominant, evidence indicates that genetic exchange between parasites has occurred. CL Brener, the cloned reference strain used for the *T*. *cruzi* genome project, is among the strains that are the products of hybridization events. More recently, genome sequences from several other *T*. *cruzi* strains have been reported [7,8], allowing comparative analyses that are essential for the understanding of the variable courses of infection and clinical forms of Chagas disease. Most importantly, the study of *T*. *cruzi* genetic diversity provides essential information for the identification of new drug targets and antigenic candidates for better diagnostic tools and vaccine development.

In addition to investigating genomic variation, differences in gene expression also provide valuable information necessary to fully understanding the complex patterns of interaction between different *T*. *cruzi* strains and their hosts. Being part of a group of early branching eukaryotes, *T*. *cruzi* possesses unique mechanisms to control the expression of its repertoire of about 12,000 genes. Similar to other trypanosomatid genomes, the *T*. *cruzi* genome is constitutively transcribed into long polycistronic transcripts containing sequences from several functionally unrelated genes. Mature, monocistronic mRNAs for protein-coding genes are produced after the pre-mRNA is processed through trans-splicing and polyadenylation, two coupled reactions determined by polypyrimidine-rich tracts located within intergenic regions (see Araújo and Teixeira, 2011 for a review [9]). As a consequence of polycistronic transcription and the scarcity of typical RNA polymerase II promoters, *T*. *cruzi* gene expression is regulated at the post-transcriptional level. Various studies have characterized regulatory sequences present in the 3’ untranslated regions (UTRs) of different genes, which act as protein-binding sites and, together with RNA binding proteins, are key elements in the control of individual mRNA steady levels during the parasite life cycle [9].

Genome-wide expression studies showing global changes in gene expression during the *T*. *cruzi* life cycle not only help to unravel the processes involved in the parasite’s interaction with different hosts but also provide an invaluable tool for the understanding of molecular mechanisms controlling gene expression in a group of early branching eukaryotes. Steady-state transcriptome analyses, based on microarray hybridizations of cDNAs derived from epimastigotes, metacyclic trypomastigote, tissue culture derived trypomastigotes and axenically-induced amastigotes of the Brazil strain show that almost 5000 genes exhibit statistically significant up or down regulation in at least one of the four life cycle stages [10]. More recently, RNA-seq analysis of the Y strain during infection of primary human foreskin fibroblasts revealed that ~15 to 30% of the predicted protein-coding genes undergo significant changes in gene expression when epimastigotes, trypomastigotes and amastigotes were compared. Focusing on the parasite energy metabolism, this study highlighted the importance of mitochondrial electron transport for intracellular survival of *T*. *cruzi* amastigotes [11]. Similar RNA-seq analysis of trypomastigotes, amastigotes and epimastigotes of the Dm28c strain showed an increased expression of genes encoding surface proteins in extracellular trypomastigotes [12]. In a comparative transcriptome analysis of *T*. *cruzi* strains belonging to the two main genetic groups present in the *T*. *cruzi* population, the Sylvio (DTU I) and Y (DTU II) strains, Houston-Ludlam et al. (2016) showed the existence of striking similarities among the differentially expressed core genes [13]. However, genome-wide analyses comparing *T*. *cruzi* strains with clear differences in their virulence properties have yet to been conducted.

Here, we present a comparative transcriptomic analysis, across multiple life cycle stages for two nearly isogenic *T*. *cruzi* cloned strains, CL Brener and CL-14, which were originally cloned from the parental CL strain [14]. In contrast to CL Brener, CL-14 is non-infectious and non-pathogenic *in vivo*, even when inoculated into newborn [15,16] or immune deficient, CD8^-/-^ mice [17] that are otherwise highly susceptible to *T*. *cruzi* infection. Importantly, after inoculation in adult animals, CL-14 prevents the development of parasitemia and mortality following challenge with the virulent CL strain [16,18,19]. Since vaccination with live CL-14 induces a potent and long-lasting parasite-specific humoral and T-cell mediated immunity against challenge with highly virulent strains of *T*. *cruzi*, the immunological adjuvant properties of the CL-14 clone is being explored as a possible vaccine vector for induction of T-cell mediated immunity against other diseases [17]. Preliminary comparisons of the CL Brener and CL-14 genomes, based on shotgun sequences, revealed only few differences in the core gene content of both strains. Although a direct comparative study between the two fully assembled genomes is still under way, our initial analyses showed that the CL-14 genome, which is also a hybrid, TcVI genome, is 99.73% identical to CL Brener (S. Teixeira, in preparation). Thus, in the absence of major differences identified so far at the genome level, we analyzed the CL-14 and CL Brener transcriptomes and compared them with the aim of identifying changes in gene expression that could explain the differences in virulence between the two strains.

## Results and discussion

### Distinct outcomes of the *in vitro* infection with CL Brener and CL-14 cloned strains

**Fig 1:**
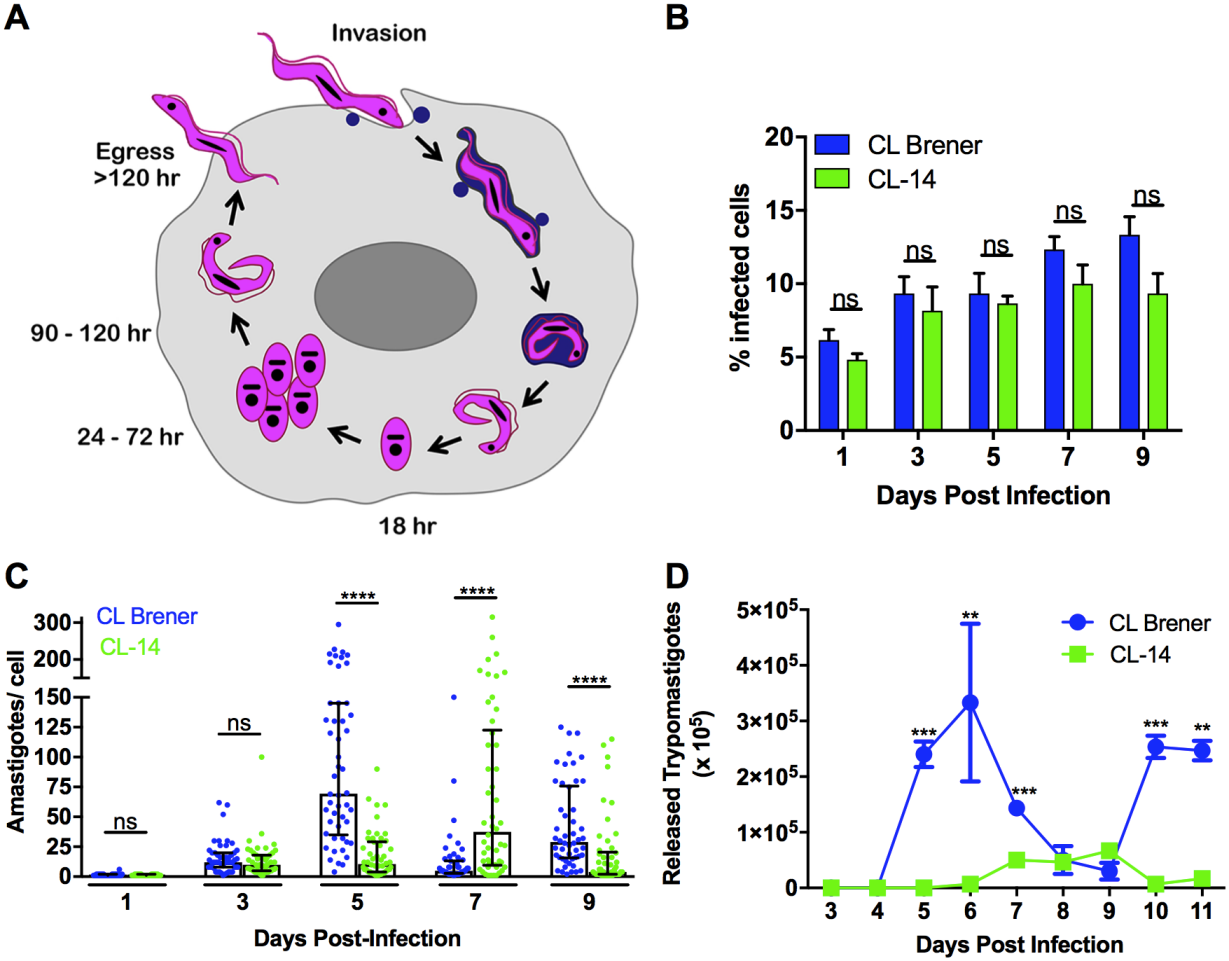
Comparative course of infection of human fibroblasts with *T*. *cruzi* CL Brener and CL-14. Intracellular *T*. *cruzi* life stages in mammalian cells: extracellular *T*. *cruzi* trypomastigotes actively penetrate mammalian cells where they differentiate into amastigotes and escape the vacuole before beginning to proliferate at ~24 hours post-infection **(A)**. Amastigotes replicate intracellularly in the host cell cytoplasm for 3–5 days, and then differentiate into motile trypomastigotes that are eventually released upon disruption of the host cell. Tissue-culture derived trypomastigotes of the CLB and CL-14 *T*. *cruzi* strains were similarly able to establish intracellular infection in cultured HFF **(B)** but exhibited markedly different intracellular growth dynamics as amastigotes. **(C)** Differences in the peak day of trypomastigote release from infected monolayers **(D)**.

The availability of two genetically similar *T*. *cruzi* strains that display marked differences in virulence presents a unique opportunity to identify fundamental phenotypic differences associated with the colonization and completion of the intracellular infection cycle in mammalian host cells (Fig 1A). To characterize the dynamics of intracellular infection by *T*. *cruzi* CL Brener and CL-14 cloned strains, parallel infections of low passage human fibroblasts (HFF) were established and monitored for several days post-infection. The capacities of CL Brener and CL-14 trypomastigotes to infect HFF were comparable with a similar fraction of the host cell monolayer colonized by these parasites (Fig 1B). In contrast, analysis of the number of intracellular amastigotes over time revealed significant differences in amastigote growth rates, with CL-14 lagging behind the faster growing CL Brener (Fig 1C). The slower growth of CL-14 amastigotes correlated with delayed release of trypomastigotes from infected cells upon completion of the intracellular growth and differentiation cycle (Fig 1D). As a consequence, significantly fewer trypomastigotes were released into the supernatant of CL-14-infected cells as compared to monolayers infected with CL Brener (Fig 1D). These findings suggest that the intracellular stages of CL-14 exhibit both growth and developmental defects, which may underlie the inability of CL-14 parasites to establish patent infection *in vivo*.

### Global transcriptome assessment from developmental stages in both strains

To further characterize the differences in host cell infection phenotype, a comparative transcriptome analysis was performed focused on the mammalian-infective stages of CL Brener and CL-14. Total RNA was isolated from extracellular trypomastigotes and from fibroblasts infected with each strain in parallel, at time points post-infection that capture active amastigote replication (60 hr) and the transition from amastigotes to trypomastigotes (96 hr) (Fig 1A). The mRNA populations derived from biological triplicates of each sample were sequenced on the Illumina platform and the total number of reads generated from all libraries as well as the percentages of reads mapped to the *T*. *cruzi* CL Brener reference genome are shown in S1 Table. Consistent with the general trend observed in the numbers of intracellular amastigotes per cell, the proportion of parasite reads mapping to the reference genome at 96 hours post-infection (hpi) was smaller in cells infected with CL-14 (12.1% on average) than with CL Brener (37.0% on average). As shown in Fig 1C, the numbers of amastigotes per cell at day 5 post infection were higher in CL Brener infected cells than in CL-14 infected cells. Also consistent with data shown in Fig 1C, a smaller difference (6.7% versus 10.7%, respectively) was observed at 60 hpi, when the numbers of intracellular amastigotes were similar between the two infections.

**Fig 2:**
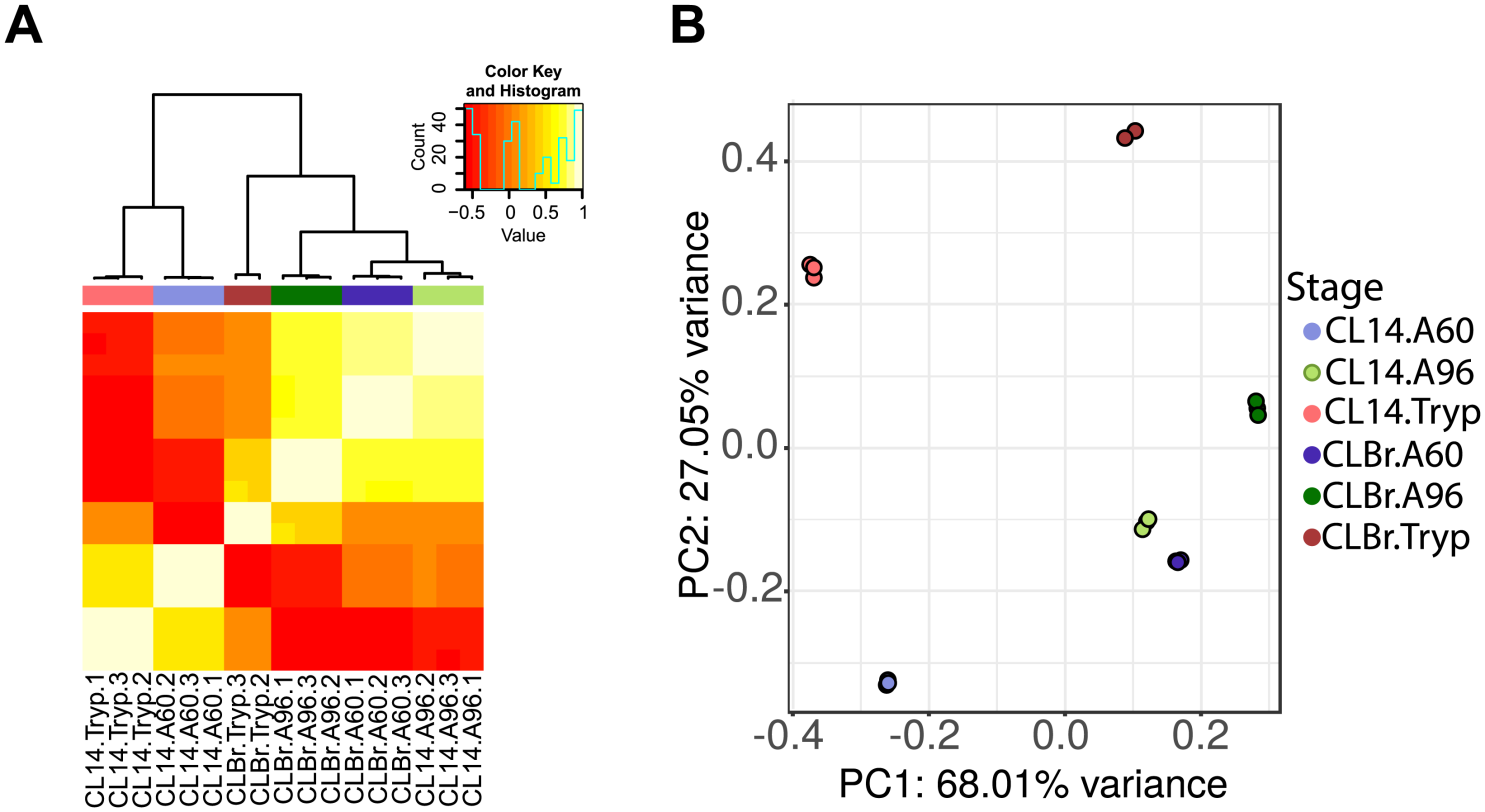
Global statistical assessment of biological replicates. Heat-map **(A)** and Principal Component Analysis (PCA) plots **(B)** of RNA-Seq data generated from the libraries mapped to the *T*. *cruzi* genome following removal of rRNA/tRNA features. Once the outlier sample was removed and data passed through normalization and surrogate variable analysis, the strong clustering by condition became evident in both analyses. In both plots, each sample is color coded by developmental stage/strain (Tryp: trypomastigotes; A60: amastigotes collected 60 hpi; A96: amastigotes collected 96 hpi).

Mapped sequencing data derived from all libraries were analyzed using a variety of methods, including principal component analysis (PCA) and hierarchical clustering, to inspect the relationships between samples and to identify potential outliers. The resulting PCA plot and heat maps (S1 Fig), after normalization, showed the expected clustering between biological triplicates with the exception of one sample generated from RNA isolated from CL Brener trypomastigotes (CLB.Tryp.1), which was identified as an outlier and excluded from subsequent analyses. Following normalization and surrogate variable analysis, the hierarchical clustering of the remaining samples revealed an intriguing set of observations (Fig 2A). The global transcriptome profiles of the CL-14 amastigotes at 96 hpi, while resembling their CL Brener counterparts, clustered tightly with the profiles of CL Brener amastigotes at 60 hpi. Additionally, the profiles from CL-14 trypomastigotes displayed a greater similarity to mammalian intracellular amastigote stages (from both strains) than the CL Brener trypomastigotes. The PCA plot displayed similar characteristics (Fig 2B). These findings suggested that a delayed expression of a large number of genes might be occurring in CL-14 amastigotes.

### Temporal patterns of surface proteins expression may play a role in parasite virulence

**Fig 3:**
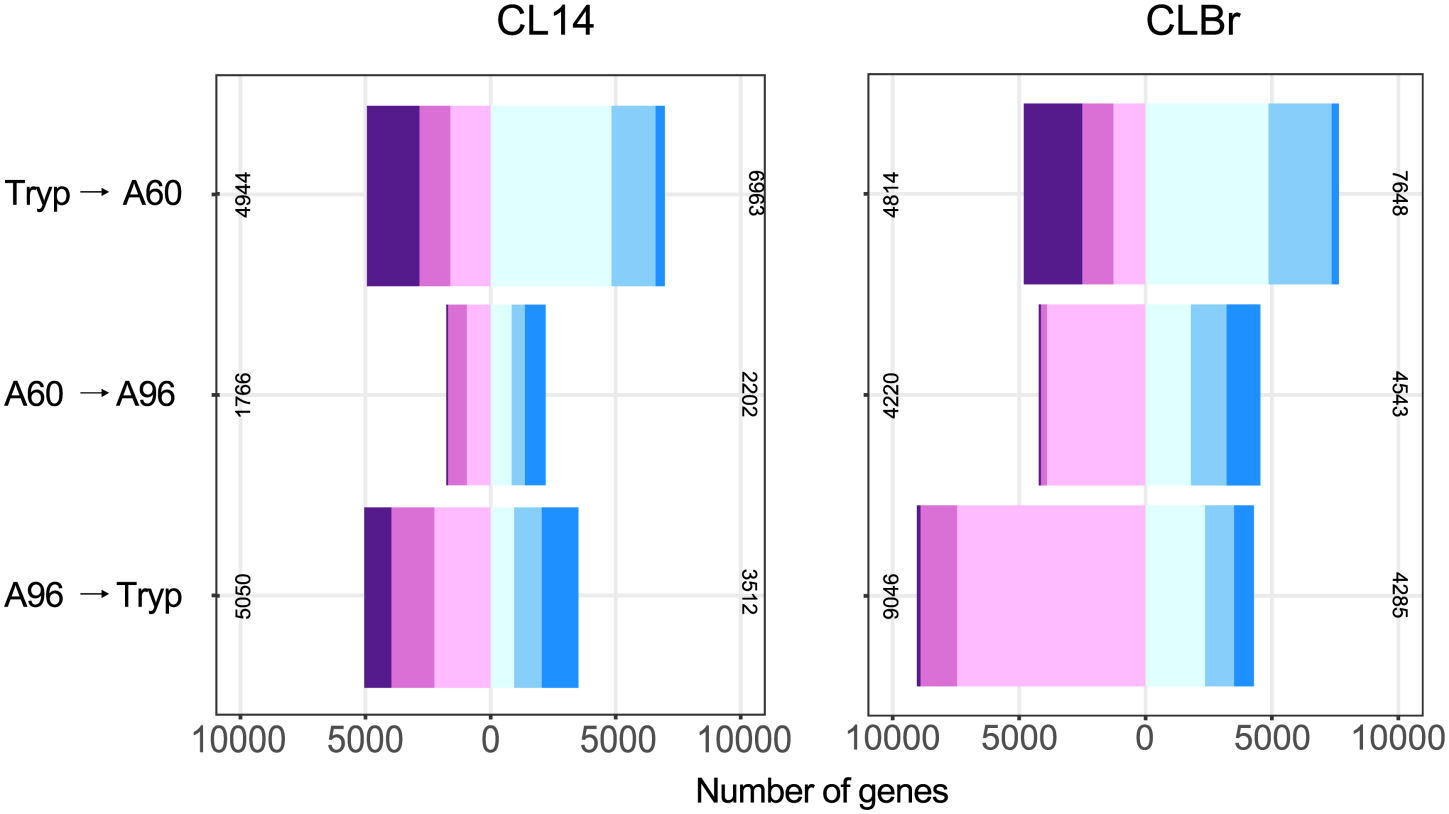
Differential gene expression across *T*. *cruzi* CL-14 and CL Brener developmental stages. Bar plots of the numbers of genes deemed significantly differentially expressed (adjusted P value <0.05) in (A) CL-14 and (B) CL Brener. The numbers of genes in each category are defined as: log2 fold changes |0–1| (light cyan for positive, light plum for negative), |1–2| (light sky blue for positive, orchid for negative), and |2+| (dodger blue and purple respectively). The diverging patterns of expression are shown by changing bar patterns.

The numbers of *T*. *cruzi* genes that emerged as differentially expressed (DE) with adjusted P value of <0.05, in pairwise comparisons of similar developmental stages of CL Brener and CL-14, are shown in Fig 3 (see S2, S3 and S4 Tables for the complete datasets). For both parasite strains, comparisons between an intracellular stage (A60 or A96) and the extracellular trypomastigote stage (Tryp) yielded a larger set of differentially expressed (DE) genes than similar contrasts between the intracellular stages (A60 vs A96) (Fig 3). This finding, which agrees with our recent characterization of global transcriptome changes in different stages of the *T*. *cruzi* Y strain parasites [11], was anticipated considering the extensive morphological and biochemical differences that exist between the intracellular and extracellular parasite life stages.

**Fig 4:**
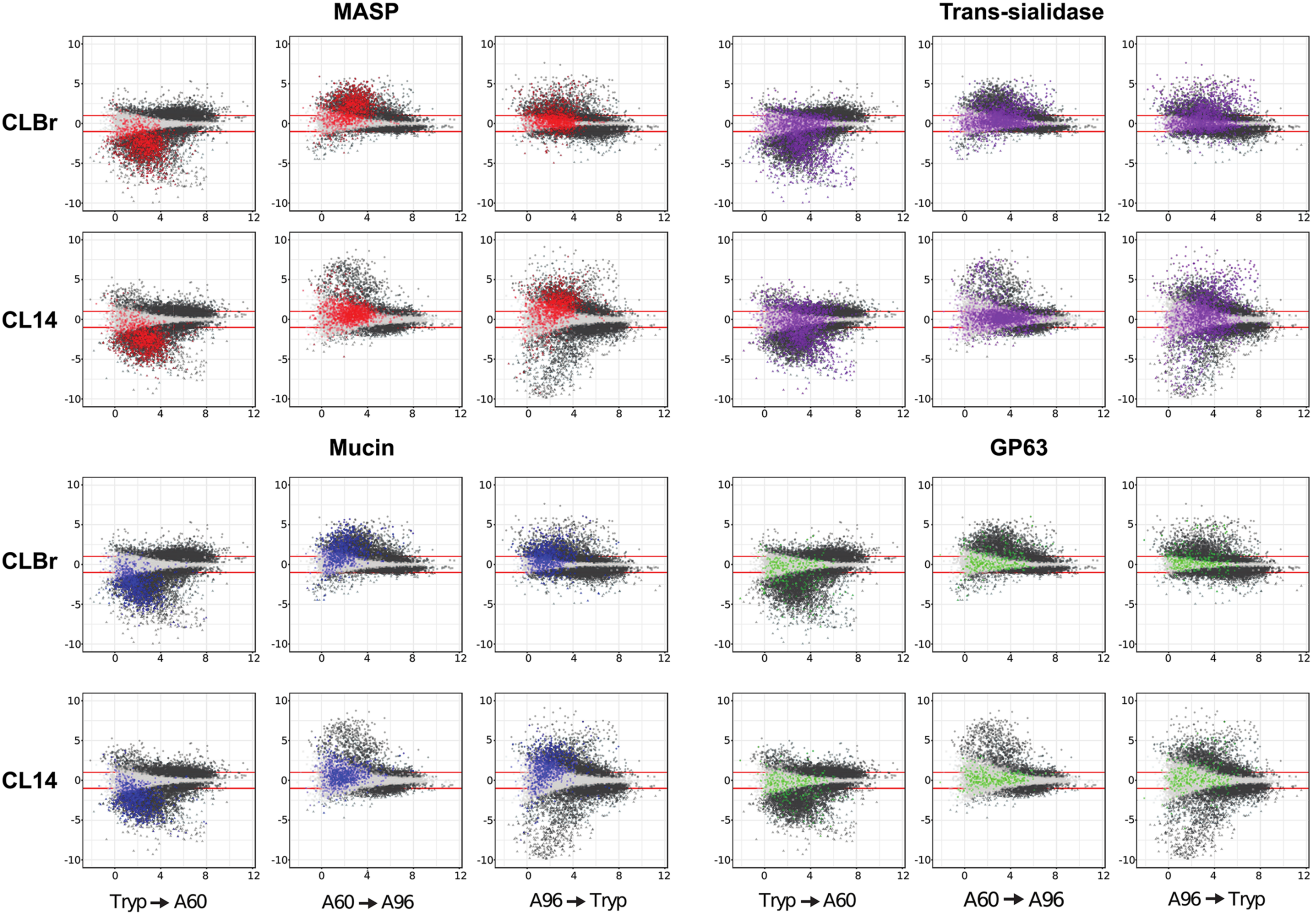
Comparative global transcriptional expression patterns of *T*. *cruzi* genes encoding polymorphic cell surface proteins in CL Brener and CL-14. MA plots depicting the log2 fold change (logFC) of genes against the average expression level during the transition of CL Brener and CL-14 across developmental stages. Each dot represents one gene and colored dots represent members of the four of the six largest *T*. *cruzi* gene families analyzed: MASP (red), Mucin (blue), Trans-sialidase (purple), and GP63 (green). Dots above and below the red lines represent differentially expressed genes (logFC >1).

As demonstrated (Fig 1B–1D), the CL Brener strain completes its intracellular life cycle in mammalian host cells more rapidly and more efficiently than CL-14: similar differences are predicted to be reflected in the global gene expression patterns. Changes in the CL Brener global transcriptome appeared to be more pronounced with twice the number of significantly up- and down-regulated genes when compared to CL-14 (Fig 3), particularly when amastigotes at 60 hpi and 96 hpi were compared. The most significant differences appeared to lie in the temporal patterns of expression of some multi-gene family encoding surface proteins, which constituted 26% to 89% of highly DE genes (>2 fold). This is best illustrated when members of multigene families of surface proteins such as trans-sialidase (TS), mucin, mucin-associated surface proteins (MASP) and GP63 were color-coded on MA plots for all three DE contrasts (trypomastigote vs. 60 hpi amastigotes, 60 hpi vs. 96 hpi and 96 hpi amastigotes vs. trypomastigotes) (Fig 4). In the contrast representing the transition of extracellular trypomastigotes to amastigotes at 60 hpi (Tryp to A60), an expected down-regulation of genes encoding TS, MASP and mucin was conspicuous for both strains and there were few differences in the behavior of most gene families in CL-14 when compared to CL Brener. The latter observation was supported by a two-sample Kolmogorov-Smirnov (K-S) test, which quantifies and tests the distance between two distributions and a Welch's test of the mean log fold changes (logFC) among the genes in each family (S5 Table). The resulting K-S distance values were small for MASP, mucin, RHS and GP63 and TS (*D* = 0.05, 0.05, 0.7, 0.09, respectively) and so were the differences in the mean logFC values. A clear and diverging pattern emerged when the selected multi-gene families were compared during differentiation from 60 to 96 hpi amastigotes. Genes encoding MASP, mucin and TS were up-regulated *en masse* as CL Brener began its transformation back to trypomastigotes while showing little change in CL-14 (*D* = 0.39, 0.35 and 0.27, respectively). A careful examination of the mean logFC change values (S5 Table) reveals a more vigorous up-regulation of MASP and mucin genes when compared to TS genes, which may reflect the roles played by these cell surface proteins during parasite development in the mammalian host. The most striking observation was a delayed up-regulation of CL-14 genes encoding MASP, mucin and TS, marking a slow transition of amastigotes to trypomastigotes when compared to CL Brener and suggesting that the processes that regulate the expression of this specific group of genes encoding parasite surface proteins may be a major factor underlying the avirulent phenotype of CL-14. It is also possible that a smaller proportion of CL-14 amastigotes differentiates into trypomastigotes, resulting in the release of fewer trypomastigotes in the extracellular milieu. Members of the DGF family displayed a similar pattern of expression in CL Brener and CL-14, however their down-regulation was considerably more pronounced as CL-14 amastigotes transform into trypomastigotes (S5 Table). It is also important to note that the clear temporal asynchronicity in expression patterns of MASP, mucin and TS genes in CL-14 when contrasted to CL Brener were not observed for members of other multigene families such as GP63 and RHS.

### Gene ontology enrichment analyses

A gene ontology (GO) enrichment analysis of DE genes in CL-14 and CL Brener revealed some trends similar to those observed in the transciptome analysis of *T*. *cruzi* Y strain [11]. Because the differentiation from trypomastigotes into intracellular amastigotes is accompanied by a massive down regulation of the expression of gene families encoding trans-sialidase, MASP and mucin surface proteins, we masked all DE genes that belong to any of the six largest multigenic families (MASP, mucin, DGF-1, Trans-sialidase, RHS and GP63) present in the CL Brener genome before performing the GO enrichment analysis. The transformation from extracellular trypomastigotes to intracellular replicative amastigotes was accompanied by enrichment of several biological processes including translation (GO:0006412), protein folding (GO:0006457), chromosome organization (GO:0051276), ion transport (GO:0006811), lipid biosynthesis (GO:0008610), and oxidoreductase activity (GO:0016491) in both parasite strains (Table 1). As discussed by Li et al. [11], these changes reflect the rapid proteome and membrane lipid remodeling as well as metabolic adaptations to distinct carbon sources that are required for parasite replication and intracellular survival. Interestingly, GO enrichment analyses described in the Y strain revealed that genes related to ion transport are among the most highly differentially expressed genes in amastigotes at 48 and 72 hpi compared to trypomastigotes. One of those genes (TcCLB.509197.39) belongs to the ZIP family of metal transporters that was also found highly expressed in 60 hpi amastigotes of CL Brener and CL-14 compared to trypomastigotes (logFC of 4.9 and 4.3, respectively). It is important to note that while both strains shared most of the same enriched GO processes, the numbers of genes that were significantly up regulated (with logFC>1) within the GO categories were consistently higher in CL Brener amastigotes than in CL-14. For example, whereas 109 genes related to oxidoreductase activity were up-regulated during the transformation of CL Brener trypomastigotes into 60 hpi amastigotes, only 58 genes were up-regulated in 60 hpi amastigotes of CL-14. Likewise, the numbers of genes involved in ion transport and lipid biosynthesis that were up-regulated in 60 hpi amastigotes were in general 50% lower in CL-14 when compared to CL Brener. These data indicate that the differentiation of non-dividing trypomastigotes into replicative and more metabolic active amastigotes may be occurring at a slower pace in CL-14 compared to CL Brener. On the other hand, when amastigotes at 60 hpi and 96 hpi are compared, the number of genes in the two GO categories that are upregulated in 96 hpi amastigotes of CL-14 are greater than in CL Brener, which also indicates a slower reprogramming of gene expression during intracellular differentiation of CL-14 compared to CL Brener. Among the two GO categories that were enriched with down-regulated genes when trypomastigotes from either strain differentiated into amastigotes, was a hexose transport biological process. In the current *T*. *cruzi* genome database only one gene encoding a glucose transporter (TcCLB.506355.10) is annotated, but after a more careful inpection, we identified two additional hexose transporter orthologues. Transcript levels of all three hexoxe transporters were significantly down-regulated in amastigotes from both strains (for TcCLB.506355.10, with a logFC -2.418 in CL Brener and -2.747 in CL-14), as expected, since carbohydrates do not constitute a significant energy source for intracellular amastigotes [20].

**Table 1:** Gene ontology (GO) categories (biological process, BP and metabolic function, MF) enriched across the *T*. *cruzi* CL Brener and CL-14 life cycles.

	GO Term (CL Brener)	*P* value	Num. DE	Total Num.
**Trypomastigote to Amastigote 60 hpi, up-regulated**				
GO:0006412	translation (BP)	2.52E-55	177	475
GO:0016491	oxidoreductase activity (MF)	6.13E-17	109	455
GO:0006811	ion transport (BP)	3.57E-16	56	165
GO:0006457	protein folding (BP)	1.87E-15	45	118
GO:0051276	chromosome organization (BP)	2.47E-12	30	70
GO:0008610	lipid biosynthetic process (BP)	5.87E-10	40	134
**Trypomastigote to Amastigote 60 hpi, down-regulated**				
GO:0004672	protein kinase activity (MF)	2.78E-04	32	418
GO:0008645	hexose transport (BP)	4.02E-02	1	1
**Amastigote 60 hpi to Amastigote 96 hpi, up-regulated**				
GO:0006565	L-serine catabolic process (BP)	3.51E-05	3	3
GO:0001539	cilium or flagellum-dependent cell motility (BP)	7.82E-03	3	12
**Amastigote 60 hpi to Amastigote 96 hpi, down-regulated**				
GO:0006457	protein folding (BP)	1.01E-05	8	118
GO:0006730	one-carbon metabolic process (BP)	2.30E-04	2	3
GO:0006811	ion transport (BP)	1.56E-02	5	165
GO:0016491	oxidoreductase activity (MF)	2.00E-02	9	455
**Amastigote 96 hpi to Trypomastigote, up-regulated**				
GO:0004672	protein kinase activity (MF)	2.13E-04	18	418
GO:0008645	hexose transport (BP)	1.67E-02	1	1
**Amastigote 96 hpi to Trypomastigote, down-regulated**				
GO:0016491	oxidoreductase activity (MF)	1.80E-15	69	455
GO:0006457	protein folding (BP)	1.97E-12	29	118
GO:0006757	ATP generation from ADP (BP)	1.41E-10	14	31
GO:0008610	lipid biosynthetic process (BP)	3.25E-10	28	134
GO:0006811	ion transport (BP)	2.71E-09	30	165
GO:0051276	chromosome organization (BP)	1.56E-08	18	70
GO:0009116	nucleoside metabolic process (BP)	9.22E-05	18	124
GO:0006412	translation (BP)	1.49E-03	41	475
	**GO Term (CL-14)**	***P* value**	**Num. DE**	**Total Num**.
**Trypomastigote to Amastigote 60 hpi, up-regulated**				
GO:0006412	translation (BP)	1.39E-73	166	475
GO:0051276	chromosome organization (BP)	3.56E-13	26	70
GO:0006811	ion transport (BP)	5.08E-07	31	165
GO:0006457	protein folding (BP)	5.11E-07	25	118
GO:0016491	oxidoreductase activity (MF)	9.90E-06	58	455
GO:0008610	lipid biosynthetic process (BP)	2.99E-03	19	134
**Trypomastigote to Amastigote 60 hpi, down-regulated**				
GO:0004672	protein kinase activity (MF)	7.48E-04	29	418
GO:0008645	hexose transport (BP)	3.68E-02	1	1
**Amastigote 60hpi to Amastigote 96 hpi, up-regulated**				
GO:0051276	chromosome organization (BP)	2.79E-04	10	70
GO:0006811	ion transport (BP)	7.65E-03	14	165
**Amastigote 60hpi to Amastigote 96 hpi, down-regulated**				
GO:0016491	oxidoreductase activity (MF)	1.40E-02	24	455
**Amastigote 96 hpi to Trypomastigote, up-regulated**				
GO:0004672	protein kinase activity (MF)	1.40E-02	30	418
GO:0008645	hexose transport (BP)	4.86E-02	1	1
**Amastigote 96 hpi to Trypomastigote, down-regulated**				
GO:0006412	translation	1.73E-47	151	475
GO:0051276	chromosome organization	8.50E-15	31	70
GO:0006811	ion transport	1.05E-11	46	165
GO:0006720	isoprenoid metabolic process	3.47E-06	11	23
GO:0016491	oxidoreductase activity	2.60E-05	71	455
GO:0006457	protein folding	1.62E-04	24	118
GO:0008610	lipid biosynthetic process	1.81E-04	27	134
GO:0006730	one-carbon metabolic process	1.00E-03	3	3
GO:0009116	nucleoside metabolic process	1.30E-03	23	124

(P-value cutoff of <0.05; Number of DE genes and total number of genes in each GO term are shown)

When we contrasted the GO enrichment analyses for the amastigotes’ transition from 60 hpi to 96 hpi in both strains, a few notable differences were observed, including a category involved with cilium or flagellum-dependent cell motility (GO:0001539), which appeared up-regulated only in CL Brener at 96 hpi. Ciliary and flagellar motility also emerged as an enriched GO terms associated with mature *T*. *cruzi* amastigotes as early as at 48 hpi in Y strain [11]. Another interesting difference from CL Brener included an enrichment of CL-14 genes involved in chromosome organization (GO:0051276) and ion transport (GO:0006811) that was sustained in CL-14 amastigotes at 96 hpi. We noted earlier that genes encoding several cell surface proteins were up-regulated *en masse* as CL Brener began its transformation back to trypomastigotes, i.e., when 60 hpi and 96 hpi amastigotes were compared. In CL-14, the induced expression of these surface gene families became evident only when 96 hpi amastigotes and trypomastigotes were compared. The GO enrichment analyses indicated that in addition to genes encoding cell surface proteins, amastigotes of CL-14 at 96 hpi did not express genes required for the last steps of amastigote-to-trypomastigote differentiation, such as genes involved with flagellum-dependent motility (TcCLB.510955.40, TcCLB.509005.10, TcCLB.508793.14). Also, genes related to protein folding (GO:0006457) and ion transport (GO:0006811), which were down-regulated in 96 hpi amastigotes compared to 60 hpi amastigotes in CL Brener did not show significant differences in gene expression when 60 hpi and 96 hpi amastigotes of CL-14 were compared.

When GO enrichment was examined for the differential expression from amastigotes at 96 hpi to trypomastigotes, an expected enrichment of protein kinase activity (GO:0004672) and hexose transport (GO:0008645) was identified amongst the upregulated genes in both strains. These finding are in agreement with data that showed that glucose is the main energy source for the parasite in the bloodstream and also with increased protein modification, such as phosphorylation of MASPs, that is required for the abundant expression of cell surface glycoproteins in trypomastigotes [12,20,21,22]. Also consistent with a decreased metabolism and macromolecular biosynthesis of the non-replicative trypomastigotes, down-regulated genes in both strains were enriched in protein translation (GO:0006412), protein folding (GO:0006457), ion transport (GO:0006811), oxidoreductase activity (GO:0016491), lipid biosynthesis (GO:0008610) and chromosome organization (GO:0051276). Hence, in addition to confirming the associations between gene expression and the metabolic differences that are known to occur during the intracellular differentiation, our GO enrichment analyses revealed further differences in gene expression between CL Brener and CL-14 during the transition from early to late stages of the intracellular amastigote development. It can be assumed that, in addition to a distinct program controlling the expression of multigene families encoding cell surface glycoproteins, other differences in gene expression related to changes in the parasite metabolism and cellular structures may also contribute to the distinct behavior of the two strains during infection of mammalian cells.

### Differential expression of RNA-binding proteins

**Fig 5:**
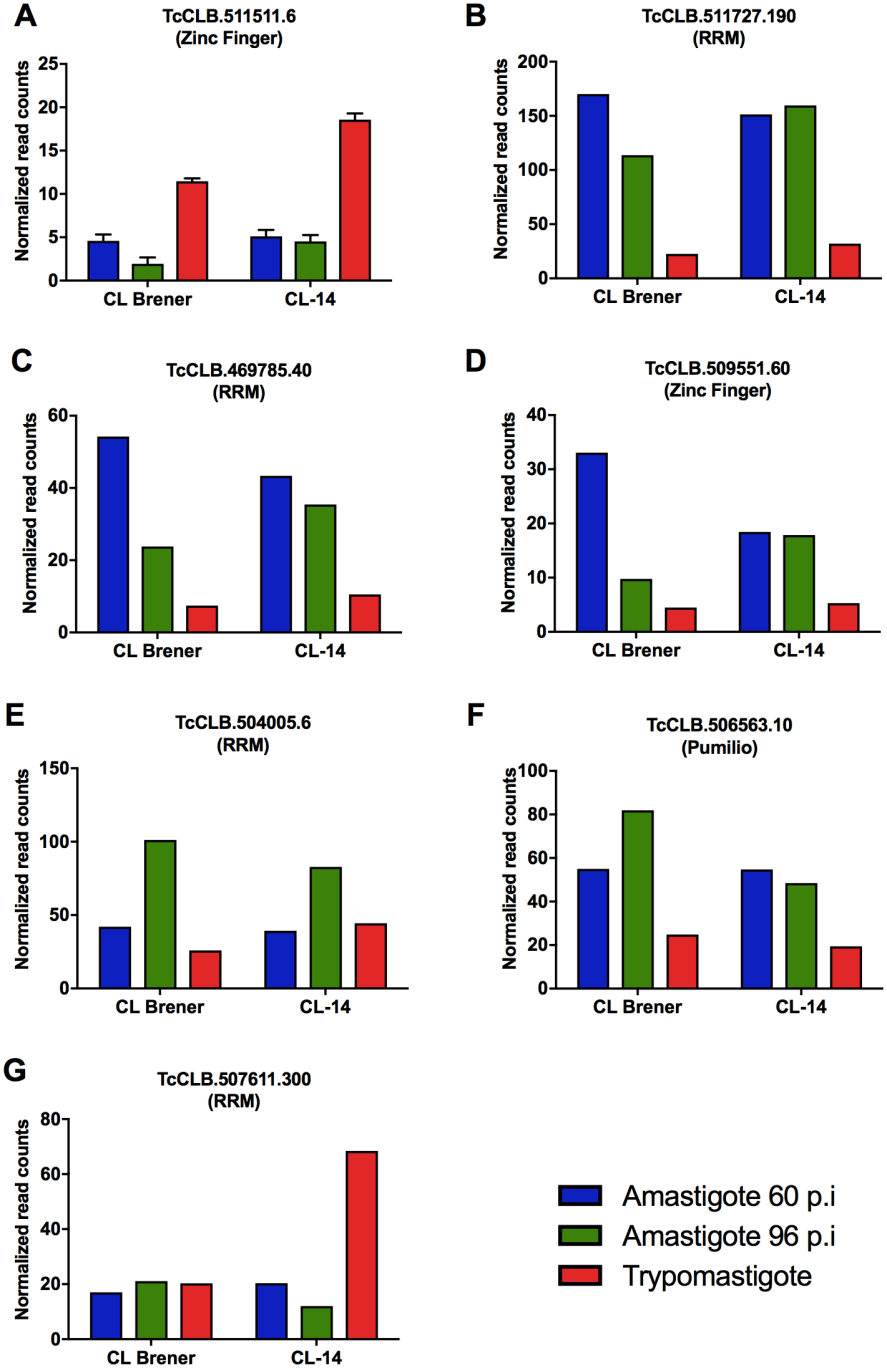
Expression patterns of selected genes encoding RNA binding proteins. Expression values for genes encoding RPBs were extracted from S4 Table. The selected genes were observed to be up-regulated in CL Brener and CL-14 trypomastigotes **(A)**, in CL Brener and CL-14 amastigotes **(B-F)**, and only in trypomastigotes from CL-14 **(G)**. Only genes encoding proteins with RNA binding domains whose transcripts present logFC > 2 comparing amastigotes at 60 hpi and 96 hpi and trypomastigote stages are shown. When sequences from both haplotypes are present, the sequences shown in the graphs correspond to Esmeraldo-like alleles, but similar expression patterns were obtained with the non-Esmeraldo alleles.

To further investigate the regulatory mechanisms controlling the distinct transcriptional programs that drive the development from intracellular amastigotes to the extracellular trypomastigote stage in the two cloned strains, we analyzed the expression of RNA-binding proteins (RBPs) that are differentially expressed throughout the infection of the host cell by CL Brener and CL-14. Using homology searches to four protein domains known to be present in RNA binding proteins (PUF (Pumilio), zinc finger, RNA recognition motif (RRM) and Alba domains), we identified a total of 253 *T*. *cruzi* sequences representing 147 genes (since two alleles were identified for most genes), which are shown in S6 Table. This list includes all genes encoding *T*. *cruzi* RBPs containing the RRM motif identified by Gaudenzi et al. (2005) [23] and Guerra-Slompo et al. (2012) [24], Pumilio-type RNA binding proteins identified by Caro et al. (2006) [25] and Zinc Finger containing proteins identified by Kramer et al. (2010) [26], with an additional 13 genes. Among all the putative RBP-encoding genes identified, six genes were differentially expressed in CL Brener (logFC>2, *P* value < 0.05) during differentiation between 60 hpi and 96 hpi amastigotes and from amastigotes to trypomastigotes (Fig 5A–5F). Amongst them is the gene encoding a CCCH zinc finger (ZFN) motif-containing protein (TcCLB.511511.6) that was significantly upregulated in trypomastigotes from CL Brener as well as in CL-14 (Fig 5A). Previous studies with the Dm28c strain of *T*. *cruzi* indicate that this RBP acts as a regulatory factor possibly involved with metacyclogenesis [27], whereas the *T*. *brucei* homologue TbZFN1 (Tb927.6.3490) was identified as a factor that is upregulated during differentiation from bloodstream to procyclic forms [28].

Five transcripts encoding RBPs are upregulated in amastigotes from both strains, indicating that they may control expression of genes involved with replication and intracellular survival of the parasite in the mammalian host cell. TcCLB.511727.190 encodes a RRM containing protein, which was significantly upregulated in 60 hpi and 96 hpi amastigotes compared to trypomastigotes of CL Brener and CL-14 (Fig 5B). Its *T*. *brucei* homologue DRBD18 (Double RNA Binding Domain, encoded by Tb927.11.14090) can be modified by arginine methylation [29]. TcCLB.469785.40, identified as amastigote-specific not only in CL Brener and CL-14 but also in the Y strain [11], is also a member of the DRBD family whose *T*. *brucei* ortholog (Tb927.6.3480) has been identified as the post-transcriptional repressor DRBD5 [30] (Fig 5C). Similar to TcCLB.469785.40, transcript levels of the RBP encoded by TcCLB.509551.60 is increased in 60 hpi amastigotes of CL Brener compared to 96 hpi amastigotes and trypomastigotes as well as in amastigotes of the Y strain up to 12 hpi [11] (Fig 5D).

TcCLB.504005.6, annotated as RBP5 (Fig 5E) encodes an RBP that is upregulated in 96 hpi amastigotes of CL Brener and CL-14, as well as in the Y strain amastigotes at 72 hpi amastigotes but not at 48 hpi [11]. Similarly, transcript levels of the *T*. *cruzi* PUF9 (TcCLB.506563.10) are also up-regulated in CL Brener 96 hpi amastigotes as well as in Y strain amastigotes at 72 hpi compared to amastigotes at early stages of the infection (Fig 5F). In *T*. *brucei*, PUF9 (Tb927.1.2600) stabilizes a group of transcripts during S-phase and may be involved with the temporal coordination of nuclear and kinetoplast replication [31]. It is therefore tempting to speculate that the RBPs encoded by TcCLB.504005.6 and TcCLB.506563.10 act as regulatory factors involved with the final steps of amastigote-trypomastigote differentiation and the release of trypomastigotes from infected cells.

Because of their critical role in regulating gene expression in trypanosomatids, we searched for RBPs whose expression significantly differs in the CL Brener and CL-14 transcriptomes. In contrast to all six differentially expressed RBP genes shown in Fig 5A–5F, TcCLB.507611.300 is constitutively expressed in the mammalian stages of CL Brener as well as in the Y strain [11], but its transcript levels are significantly increased in trypomastigotes of CL-14 (Fig 5G). TcCLB.507611.300 encodes a spliceosomal protein that is part of the U1A small nuclear ribonucleoprotein complex as indicated by the studies with the *T*. *brucei* ortholog (Tb927.10.830) [32]. In addition to further investigating whether the differences observed in the expression of CL Brener and CL-14 RBP genes, such as TcCLB.469785.40 and TcCLB.509551.60, which may be involved with the control of steady state levels of their target transcripts, further studies are also needed to verify whether the differential expression of an RBP involved in mRNA processing may cause any impact on the expression of genes related to the virulence of CL-14 trypomastigotes.

### Constitutive expression of a trans-sialidase gene in CL-14 affects amastigote to trypomastigote differentiation

Because data shown in Fig 4 and S5 Table indicated that several genes encoding surface protein families are been expressed at later time points during intracellular infection of CL-14 compared to CL Brener, we sought to test whether altering the expression of a member of these multigene families might affect parasite differentiation inside the host cell. We selected the trans-sialidase (TS) gene Tc00.1047053509495.30 (whereby named TS95.30) because it belongs to TcS group I and encodes an enzymatically active TS that contains the C-terminal SAPA (Shed Acute Phase Antigen) repeats known to be expressed only in the mammalian stages of the parasite life cycle [33]. Our RNA-seq analyses confirmed the expression of TS95.30 in amastigotes and trypomastigotes of CL Brener and CL-14 but also highlighted distinct expression levels of TS95.30, which is highly upregulated (FC = 9.8) in 96 hpi amastigotes compared to 60 hpi in CL Brener, and less so in CL-14 (FC = 3.5).

**Fig 6:**
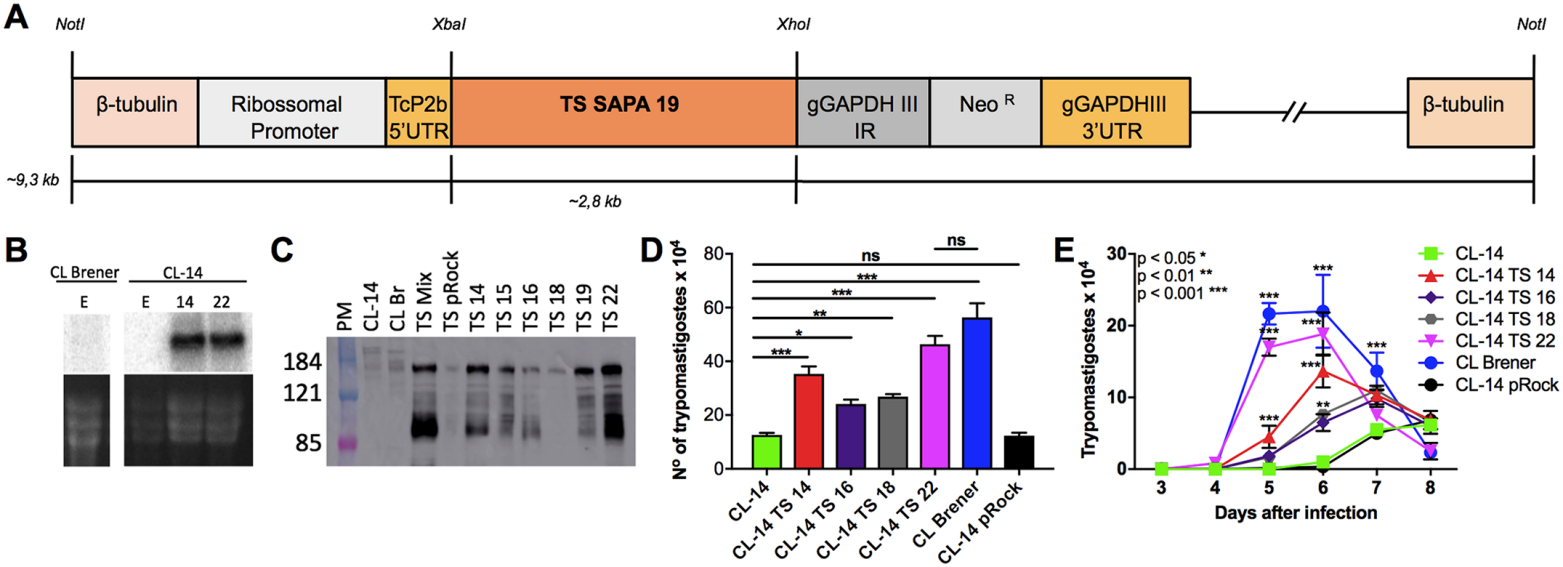
Constitutive expression of a TS gene in CL-14. The pROCKNeo vector used for transfection of CL-14 has the TS gene (Tc00.1047053509495.30) flanked by the ribosomal promoter and sequences containing signals for mRNA processing derived from the constitutively expressed housekeeping genes TcP2β (at the 5’ end) and gapdh (at the 3’ end) **(A)**. Total RNA purified from epimastigotes from WT and transgenic parasites were subjected to northern blot and hybridized with a ^32^P-labelled probe that contains sequences corresponding to the C-terminal SAPA repeats present in the TS gene. Lower panel shows ethidium bromide staining of rRNAs in the same gel before transferring to the membrane **(B)**. Total protein extracts from epimastigotes from WT and transgenic parasites were evaluated for the expression of the transfected TS gene by western blotting with a monoclonal antibody anti-SAPA **(C)**. The infection profiles of four cloned cell lines derived from CL-14 parasites transfected with the TS gene or with the empty pROCKNeo vector, were compared to WT CL-14 and CL Brener in *in vitro* infection assays of Vero cells. Equal numbers of tissue culture derived trypomastigotes from each parasite cultures were added to Vero cell monolayers and the total number of trypomastigotes released in the supernatant **(D)** or the numbers of trypomastigotes released in the supernatant each day post-infection were evaluated over 8 days **(E)**. Five replicates for each infection experiment were performed.

The complete open reading frame of TS95.30 was cloned in the pROCKNeo vector [34] and transfected into CL-14 epimastigotes using the linearized form of the vector shown in Fig 6A. As described previously, transfection with linearized pROCKNeo vector results in the integration of the DNA construct in the tubulin locus and, because signals for mRNA processing of the foreign gene are derived from the constitutively expressed housekeeping genes *TcP2*β (at the 5’ end) and *gapdh* (at the 3’ end), the pROCKNeoTS transgene can be constitutively expressed in all parasite life cycle stages, including epimastigotes [34]. After selecting a neomycin-resistant population as well as transfected cloned cell lines, total RNA and protein extracts from these cultures were subjected to northern (Fig 6B) and western blot analyses (Fig 6C). For northern blot, we used a probe derived from the 3’ region of the TS95.30 gene and for the western blot we used an anti-SAPA antibody that detects the expression of members belonging to TS group 1 sub-family. As shown in Fig 6B, two cloned epimastigote cell lines derived from G418-resistant, CL-14 parasites transfected with the TS gene (clones TS 14 and TS 22) showed increased levels of TS mRNA compared to untransfected CL-14 or with epimastigotes from CL Brener. These two transfected cell lines plus two additional ones (TS 16 and TS 18), all of them showing increased TS protein expression compared to WT CL-14 (Fig 6C), were selected and tested in *in vitro* infection assays. No differences in the growth curves of epimastigote cultures from all four transfected cell lines compared to WT CL-14 and CL Brener epimastigotes were observed (S2 Fig).

The infection capacity of the transfected parasites was tested *in vitro* by incubating Vero cells with equal numbers of tissue culture derived trypomastigotes from WT CL Brener, CL-14 and from the CL-14 transfected cell lines (TS 14 and TS 22) constitutively expressing TS95.30. As shown in Fig 6D and 6E, parasites from all four cell lines transfected with TS displayed faster kinetics of trypomastigote release in the supernatant compared to untransfected CL-14 or with CL-14 parasites transfected with the empty vector. Although no significant differences in the numbers of intracellular amastigotes were observed, the numbers of trypomastigote released by cells infected with transfected CL-14 parasites was increased compared to cells infected with WT CL-14 or with CL-14 transfected with the empty vector. This result indicates that the constitutive expression of one member of the TS gene family is sufficient to alter the differentiation from amastigote to trypomastigotes, a process that occurs intracellularly before trypomastigotes are released to infect new host cells. Although constitutive TS expression facilitates intracellular amastigote to trypomastigote differentiation and parasite egress from infected cells, constitutive expression of TS is not sufficient to restore parasite virulence. When the two cloned, transfected CL-14 cell lines TS 14 and TS 22 were inoculated into BALB/c mice, similar to WT CL-14 infected animals, no parasites were detected in the bloodstream at any time point after infection (S3 Fig). It should be noted that, cells infected with one transfected cell line, TS 22, showed a total number of trypomastigotes released in the supernatant that was statistically equivalent to the number of released trypomastigotes in CL Brener infected cells (Fig 6D). It is therefore assumed that differences regarding the gene expression profiles of trypomastigotes from both strains also contribute to determine the distinct virulence phenotypes observed during infection *in vivo*. The mechanisms by which the increased expression in CL-14 of a TS gene affects amastigote to trypomastigote differentiation need to be investigated. The work of Rubin-de-Celis and colleagues, [35] suggests that sialic acid present on lysosomal membrane glycpoproteins are targets of TS, since over-expression of TS in the Y strain facilitates escape of amastigotes from the parasitophorous vacuole. It is also possible that the constitutive expression of TS triggers other changes in gene expression or modifications at the cell surface that affect parasite differentiation. It has been shown that glycosylphosphatidylinositol anchored proteins such as TS, mucins and MASPs are transported to the parasite surface through the contractile vacuole complex, in a trafficking process that involves the small GTPase protein Rab11 [36]. Therefore, the possibility that early overexpression of TS in CL-14 may cause changes in membrane trafficking in a way that affects membrane remodeling events required for amastigote to trypomastigote differentiation, should also be considered. These hypotheses are currently being investigated using RNA-seq analyses. As predicted by the differential expression and GO analyses, adequate expression of mucins, MASPs and/or several other genes may also be needed during late stages of intracellular development of the parasite, along the expression of TS genes, to restore CL-14 virulence in animal models of infection. Our results also suggest that a genome wide DNA manipulation strategy allowing the inspection of multiple factors will be necessary to fully address the question regarding differences in the virulence phenotype among the two *T*. *cruzi* strains.

### Concluding remarks

Our *in vivo* infection assays confirmed previous studies, which have shown that, in contrast to an infection with the CL Brener clone, no patent parasitemia can be detected in immune competent animals that were inoculated with trypomastigotes from the attenuated CL-14 clone. However, *in vitro* infection assays showed that CL-14 trypomastigotes are able to infect and multiply within different cell types (this study tested infection of HFF and Vero cells), although the CL-14 infection resulted in lower numbers of intracellular amastigotes during early time points and a markedly reduced number of trypomastigotes released by the CL-14 infected cells compared to the CL Brener infection. One might conclude that a deficient capacity of CL-14 amastigotes to differentiate into trypomastigotes is a key factor accounting for the CL-14 attenuated phenotype. The results from the *in vitro* infection assays prompted us to investigate the molecular basis of the attenuated phenotype of CL-14 by performing gene expression analyses at two time points of the intracellular development of the two strains. As shown previously [11], once reaching the cell cytoplasm, CL Brener amastigotes replicate for about 4 days, before they differentiate back into trypomastigotes and rupture the cell. Our comparative transcriptome analyses showed that the transition of extracellular trypomastigotes to amastigotes at 60 hpi was accompanied by only few differences in gene expression between the two parasite strains, including a similar down-regulation of multi-gene families encoding TS, MASP and mucin. In contrast, large differences in the transcriptome remodeling were observed between the two strains when gene expression at 60 hpi and 96 hpi were compared, particularly regarding the expression of gene families encoding surface proteins, which are up-regulated with faster kinetics in CL Brener than in CL-14. Thus, the RNA-seq data presented in this study point to a central role of this group of proteins in the process of differentiation from the replicative amastigote to infective trypomastigotes and establishment of infection in the mammalian host.

Our RNA-seq analysis is also consistent with experimental data that shows no differences in the parasite’s invasion capacity or ability to multiply in the cytoplasm of the mammalian cell. As observed during *in vivo* infection, it is possible that CL-14 amastigotes are able to multiply in a limited number of tissues of the infected animal at least during the initial stages of the infection, but the delayed expression of genes encoding surface proteins prevents the release of trypomastigotes in the bloodstream and in neighboring tissues. This is consistent with several reports showing that, although infection with CL-14 does not result in patent parasitemia, it results in efficient immunity against a lethal challenge with virulent parasites [18,19], or against a human tumor antigen if this antigen is expressed by the parasite [17]. Because CL-14 retains its capacity to multiply during the first round of infection after trypomastigote inoculation, this initial multiplication within host tissues may be sufficient to induce a highly efficient immune response that includes a balanced Th1/Th2 cytokine production that is beneficial for the host [19].

In one study addressing the basis of the non-virulent phenotype, Atayde and co-workers [37] showed that metacyclic forms of the CL strain and from the CL-14 clone express comparable levels of gp82, a member of the TS family, and gp35/50, a member of the mucin family of glycoproteins. Although we did not analyze the gene expression profile of metacyclic forms, the RNA-seq data obtained for trypomastigotes from CL Brener and CL-14 indicated that once the parasites differentiated into trypomastigotes, their gene expression profiles still showed large differences, particularly regarding genes belonging to the multi-gene families of surface proteins. In another study where the number of extracellular trypomastigotes was determined after infection of CHO-K1 cells with CL-14, Tonelli and co-workers [38] found that increased numbers of trypomastigotes are released in the supernatants if cells are kept at 33°C instead of 37°C. In addition, the numbers of trypomastigotes released into the medium were 2.5 to 3-fold higher if the cultures were supplemented with 20 or 200 microM proline. We confirmed the increased capacity of CL-14 to differentiate from amastigote to trypomastigote if the temperature is lowered from 37 to 33°C (S4 Fig). The thermo-sensitive character of the differentiation process of CL-14 will be investigated using RNA-seq data collected at both temperatures. Using RNA-seq analyses, we are also planning to investigate whether the constitutive expression of one member of the trans-sialidase in CL-14 affects the expression of other genes that may also be involved with the amastigote to trypomastigote differentiation. Finally, RNA immunoprecipitation analyses using tagged versions of the three RNA binding proteins (TcCLB.507611.300, TcCLB.506563.10 and TcCLB.469785.40), identified as being differentially expressed in both strains, are also underway with the aim of identifying their target genes, which may correspond to genes directly involved with the *T*. *cruzi* differentiation program. However, the lack of a fully assembled genome poses a major limitation that needs to be resolved before we can perform a conclusive comparative analysis between these strains. Efforts towards the generation of the complete sequences of all chromosomes from CL Brener and CL-14 are underway using the long read sequencing technology by PacBio. With this new sequencing strategy we should be able to resolve all the complex regions in this parasite genome, particularly regions where large multigene families are present. Together with additional gene expression analyses, further genome studies investigating gene copy number variation will help understand the diversity of the *T*. *cruzi* population and its impact on parasite infection.

## Materials and methods

### Parasite cultures, RNA extraction and cDNA sequencing

The two cloned strains of *Trypanosoma cruzi*, CL Brener and CL-14 were provided by Egler Chiari, from the Federal University of Minas Gerais. Mammalian-infective stages of *T*. *cruzi* were maintained by weekly passage in monkey kidney cell line LLc-MK2 (ATCC CCL-7) grown at 37°C and 5% CO_2_ [11]. Trypomastigotes collected from infected monolayers were centrifuged for 10 min at 1,700*g* and then allowed to swim up from the pellet for 4 hrs before collecting the pure highly motile fraction in the supernatant and washing in Dulbecco's Modified Eagle Medium (DMEM) supplemented with 2% fetal bovine serum (DMEM-2). For RNA-Seq studies, experimental infections were established in human foreskin fibroblasts (HFF; ATCC CRL-2522) by exposing sub-confluent monolayers to purified trypomastigotes (80 parasites: host cell) for 2 hours at 37°C, 5% CO_2_ followed by rinsing in PBS 3–5 times to remove remaining extracellular parasites to achieve infection of 60–70% of host cells. Total RNA was extracted from infected HFF at 60 and 96 hours post-infection and from tissue culture derived trypomastigotes released in the supernatants of infected HFFs using the Trizol reagent (Invitrogen, CA, USA). After DNAse treatment, the RNA was further purified using the Qiagen RNeasy mini kit, poly(A)+ RNA was selected using oligo-dT following Illumina TruSeqv2 instructions and its integrity was assessed using an Agilent 2100 bioanalyzer. cDNA libraries were constructed using a TruSeq RNA Sample Prep Kit Version II (Illumina) with an average insert size of ~300 nt. Libraries were sequenced on an Illumina HiSeq 1500.

### Data quality assessment and visualization

Quality assessment of 100 nucleotide paired-end reads was performed via an initial evaluation with FastQC (http://www.bioinformatics.babraham.ac.uk/projects/fastqc/) and biopieces (http://biopieces.org). Illumina sequencing adapters and low quality sequences were removed with Trimmomatic [39]. The remaining sequences were mapped separately against the Esmeraldo haplotype of *T*. *cruzi* CL Brener genome, non-Esmeraldo haplotype, and a combination of both haplotypes along with the unassigned contigs using the TriTrypDB version 9.0 genome and annotation data (http://tritrypdb.org/common/downloads/release-9.0/). Alignments were done using TopHat2 [40] version 2.0.14 and options allowing a single randomly placed mapping per read (-g 1) and without a novel splice junction search (-G gff_file). Mapping was performed with the same options for the samples collected from human cell samples using the ensembl GRCh37.62.v3 release. Alignments were compressed, sorted, and indexed using samtools [41] in order to include only proper pairs and counted against the set of annotated transcripts using HTSeq [42]. The resulting count tables were passed to R for outlier testing, sample clustering, visualization, and differential expression analyses with the hpgltools package (http://github.com/elsayed-lab/hpgltools/). A table with raw counts for all samples and conditions is available as supplementary data (S7 Table).

### Differential expression and gene ontology analyses

Samples were tested for significant outliers using a mix of hierarchical clustering and principal component analysis. Non-expressed and weakly expressed genes, defined as having <2 reads per million in n of the samples, where n is the size of the smallest group of replicates (here n = 2), were removed prior to differential expression (DE) analysis. Of the 24,887 genes analyzed, 23,191 remained after applying the low-count filter. Multiple data normalization methods were tested, including a mix of: trimmed median of M-values, quantile, log transformation, and counts per million. Surrogate variables / batches were queried with a mix of methods from sva [43] and ruv [44]. Differential expression analyses were performed and compared using surrogate estimates from svaseq with limma [45], DESeq2 [46], EdgeR [47], and a statistically uninformed basic method as a diagnostic control. Genes were considered ‘significant’ when the limma observed |log2 fold-change| was greater than 1.0 (logFC>1) and the adjusted *P* value was less than 0.05. The set of significant genes for each contrast of interest was passed to Goseq [48] for the parasite samples and g:Profiler [49] for the human samples through its R interface.

### Parasite transfection and characterization

Transfected CL-14 epimastigotes were obtained by cloning the complete open reading frame (ORF) of the TS gene Tc00.1047053509495.30 into the XbaI-XhoI sites of pROCKNeo plasmid [34]. *T*. *cruzi* epimastigotes were transfected, selected with 200 microg/mL of G418 and several G418 resistant cloned cell lines were generated as previously described [17,34]. Expression of TS95.30 was detected by northern blot using a ^32^P labelled PCR fragment corresponding to the C-terminal domain of the transfected gene and by western blot using an anti-TS-SAPA monoclonal antibody (kindly provided by Sergio Shenkman, Federal University of São Paulo) according to previously described protocols [17]. *In vivo* infection of BALB/c mice (purchased from Biotério Central, Federal University of Minas Gerais) were carried out as previously described [17]. *In vitro* infection of Vero cells (ATCC CCL-81) with tissue culture derived trypomastigotes of transfected parasite cell lines were done as indicated in the previous session.

#### Ethics statement

Animal infections were performed in strict accordance with the Brazilian laws regarding animal use (LEI No 11.794, DE 8 DE OUTUBRO DE 2008). All protocols were approved by the Committee on the Ethics of Animal Experiments of UFMG (protocol # 132/2014).

## Supporting information

S1 Fig Quality control metrics suggesting the removal of one sample. (A) Raw library sizes were plotted to check for comparable sequencing depths for all samples, (B) boxplot of the count distributions by gene. Hierarchical clustering analyses using Pearson correlation (C) and Euclidean distances (E) along with Principal Component Analyses (D) and a standard medium Pearson correlation between each sample (F) of the log2, cpm, quantile, and low-count filtered data suggested the removal of sample ‘CLB.Tryp.1’.
(EPS)

S2 Fig Growth curves of CL-14 epimastigotes transfected with a TS gene. Wild-type (WT) CL-14 epimastigotes and cloned cell lines derived from CL-14 epimastigotes transfected with the pROCK vector containing the TS gene TS95.30 (TS 14, TS 16, TS 18 and TS 22) as well as CL-14 epimastigotes transfected with the empty pROCK vector were cultivated in LIT medium for 9 days and the numbers of parasites were determined in a Neubauer chamber.
(TIFF)

S3 Fig Infection of BALB/c mice with WT and transgenic CL-14. Groups of 5 animals were inoculated with 5 x 10^3^ tissue culture trypomastigotes from WT CL-14, from transgenic CL-14 cell lines TS 14 and TS or from CL Brener trypomastigotes and parasitemia was determined during 30 days. The data shown are representative of three independent experiments.
(TIFF)

S4 Fig Cell infection assays with CL-14 trypomastigotes at different temperatures. Vero cells (5 x 10^4^) were incubated with 5 x 10^5^ CL-14 trypomastigotes at 37° or 34°C and 5% CO_2_. After 24 hours, cells were washed with phosphate buffered saline to eliminated extracellular parasites and the numbers of trypomastigotes released in the supernatant were determined in a Neubauer chamber. Infection assays were performed in triplicates.
(TIF)

S1 Table Summary of samples collected, mapping statistics and experimental metadata. This summarizes the experimental reference identifiers used in the experiment (columns A-C), the experimental conditions (columns D-F), experimental batches (column G), numbers of reads collected and mapped (columns H-J), and percentage mapped to the parasite and host genomes (columns K-N).
(XLSX)

S2 Table Differential expression for all contrasts and all genes using limma. The workbook contains one worksheet for every contrast performed by limma, starting with the annotation information provided by the TriTrypDb, followed by the limma statistics for every coding-sequence gene which passed low count filtering. A detailed legend is provided in the first worksheet.
(XLSX)

S3 Table Lists of significant differentially expressed genes for all contrasts using limma. This workbook contains the same information as in Table S2, but only for those genes which are deemed ‘significantly differentially expressed’ using a |log2 fold change| > = 2.0 and adjusted p-value < = 0.05. The worksheets are named according to the contrast performed, and the direction of the fold change. The sheet ‘number_changed’ provides some summary information regarding the numbers of observed differentially expressed genes according to each tool employed.
(XLSX)

S4 Table Expression values for individual genes. The metric of ‘expression’ was collected from limma following the application of voom for each experimental condition. It is comprised of the normalized, batch-adjusted, expression value on the log2 scale. Errors were calculated by taking the logFC from the limma identity tables and dividing by the t-statistic for each gene. The table contains annotations (columns A-E), followed by the metric of expression for each condition (columns F-K), followed by the standard error (columns L-Q) for each condition.
(XLSX)

S5 Table Analysis of expression trends in six *T*. *cruzi* multigene families. A two-sample Kolmogorov–Smirnov test (K–S test) and a Welch’s two sample t-test for unequal variances were run to test for significant differences in the distributions (K-S) and means (Welch’s) of the logFC values for all members of each multigene family across development.
(XLSX)

S6 Table Putative RNA-binding domain containing genes. Genes were identified using homology searches to four protein domains known to be present in RNA binding proteins: PUF (Pumilio), zinc finger, RNA recognition motif (RRM) and Alba domain. For most genes, the two alleles (Esmeraldo-like and non-Esmeraldo-like) are shown.
(XLSX)

S7 Table Raw counts for all samples. The workbook comprises 7 sheets. The first, 'legend', contains a legend and copy of the experimental design. The 'raw_reads' sheet contains the gene annotation data along with numbers of raw counts observed for each gene. The 'raw_graphs' a series of initial plots describing the raw data. The 'norm_data' provides the counts after the normalization applied as stated in the legend (log2, quantile, sva, filtered). The 'norm_graphs' provides the same set of plots describing the normalized data. Finally, the 'median_data' provides a set of median values of the data after normalization by experimental conditions.
(XLSX)
