# Supplementary figures and images for: Comparative transcriptome profiling of virulent and non-virulent Trypanosoma cruzi underlines the role of surface proteins during infection
Source: PLoS Pathog. 2017 Dec 14;13(12):e1006767. doi: 10.1371/journal.ppat.1006767 (PMC5746284; doi:10.1371/journal.ppat.1006767)

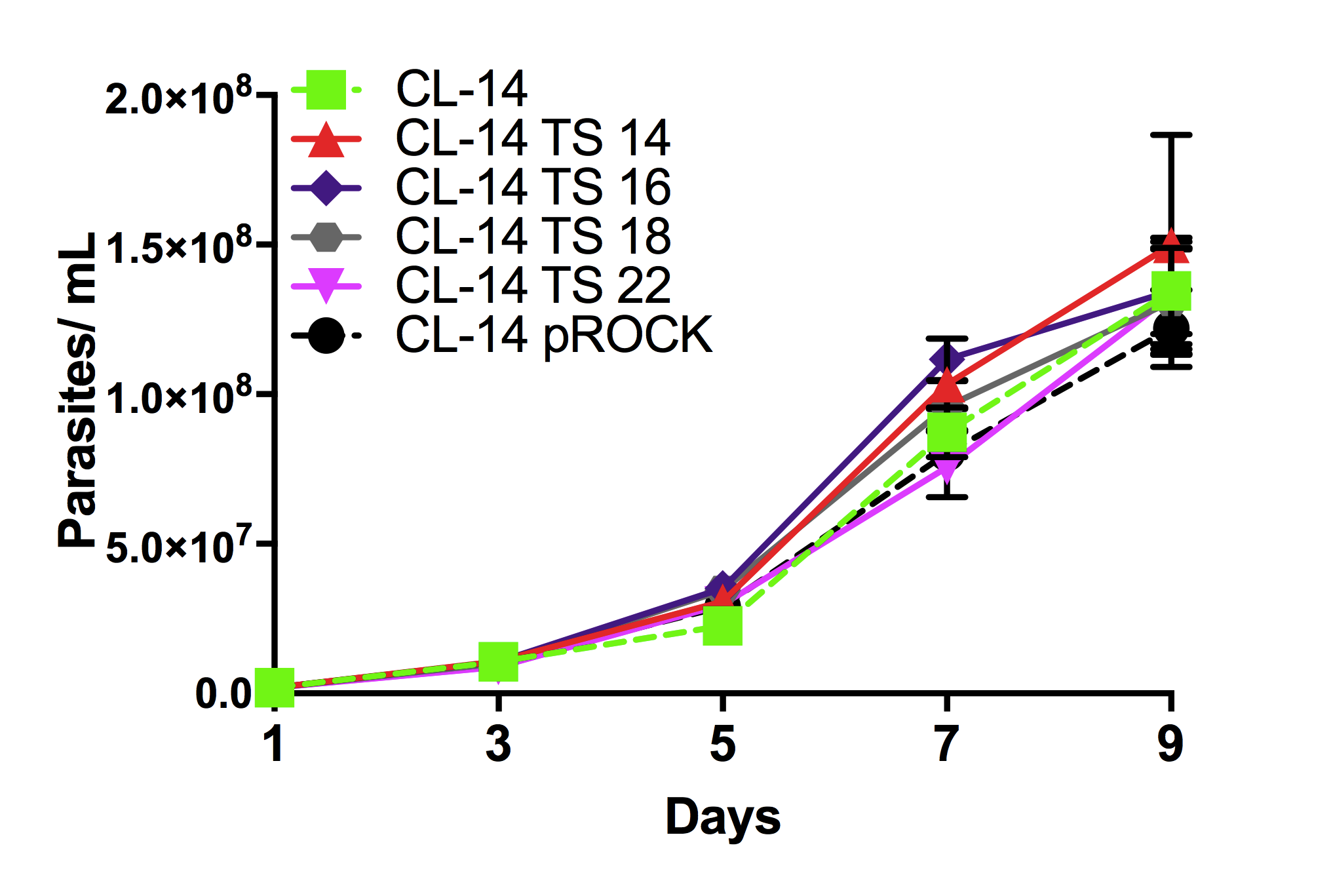

Supplement: S2 Fig — Wild-type (WT) CL-14 epimastigotes and cloned cell lines derived from CL-14 epimastigotes transfected with the pROCK vector containing the TS gene TS95.30 (TS 14, TS 16, TS 18 and TS 22) as well as CL-14 epimastigotes transfected with the empty pROCK vector were cultivated in LIT medium for 9 days and the numbers of parasites were determined in a Neubauer chamber. (TIFF) [file ppat.1006767.s002.tiff]

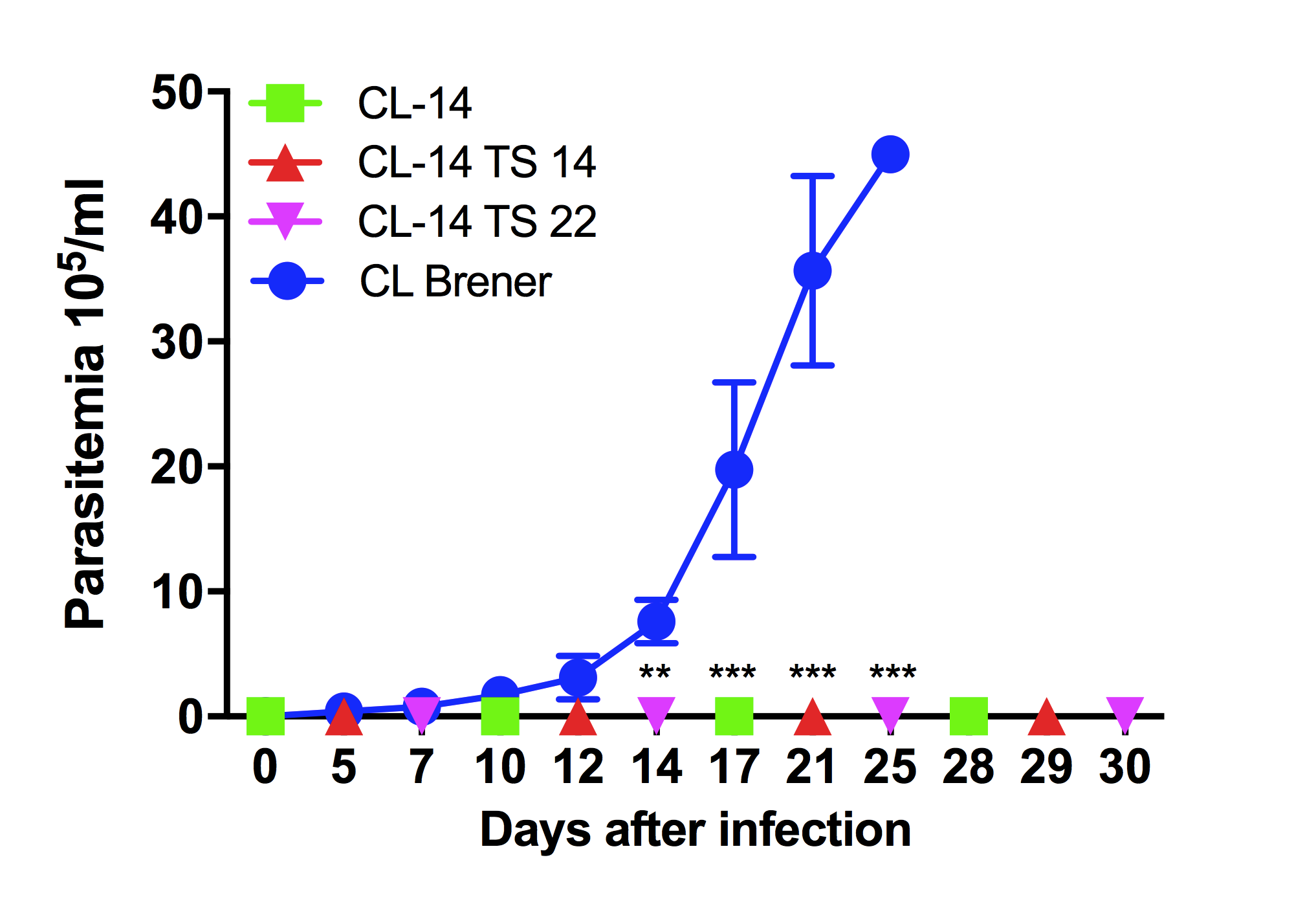

Supplement: S3 Fig — Groups of 5 animals were inoculated with 5 x 10^3^ tissue culture trypomastigotes from WT CL-14, from transgenic CL-14 cell lines TS 14 and TS or from CL Brener trypomastigotes and parasitemia was determined during 30 days. The data shown are representative of three independent experiments. (TIFF) [file ppat.1006767.s003.tiff]

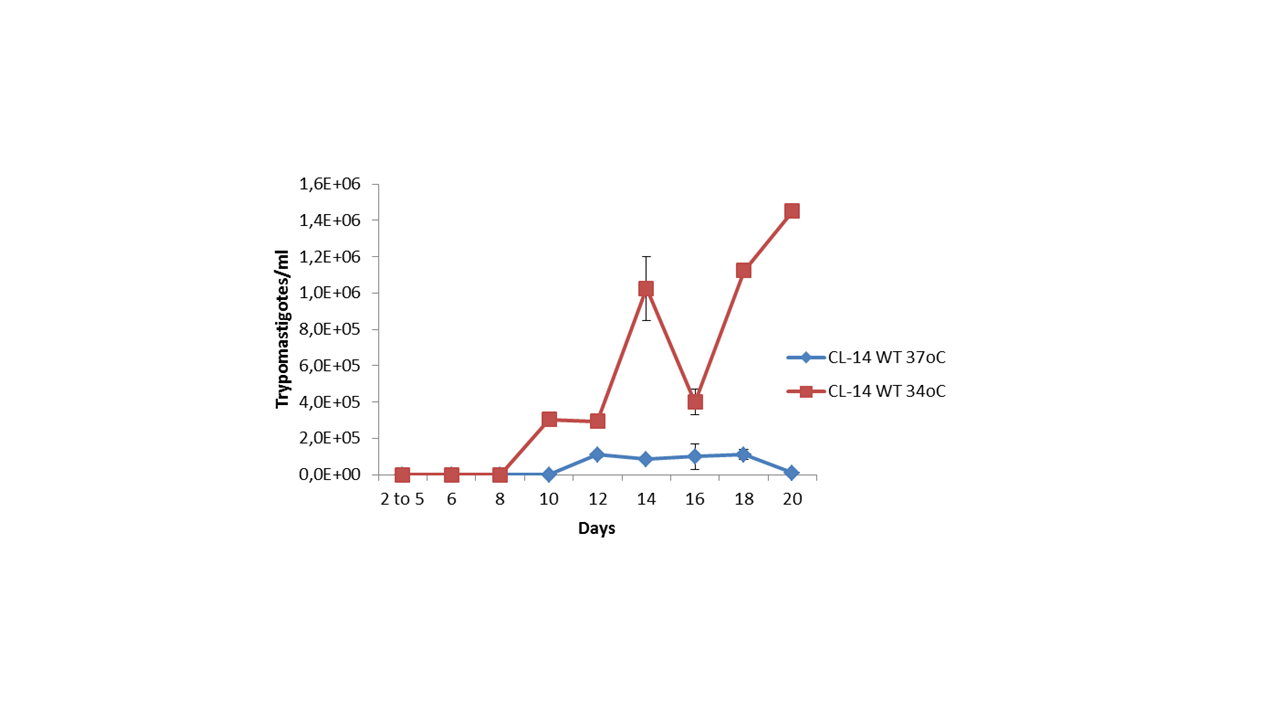

Supplement: S4 Fig — Vero cells (5 x 10^4^) were incubated with 5 x 10^5^ CL-14 trypomastigotes at 37° or 34°C and 5% CO2. After 24 hours, cells were washed with phosphate buffered saline to eliminated extracellular parasites and the numbers of trypomastigotes released in the supernatant were determined in a Neubauer chamber. Infection assays were performed in triplicates. (TIF) [file ppat.1006767.s004.tif]
